# The LaMIT database: A read speech corpus for acoustic studies of the Italian language toward lexical access based on the detection of landmarks and other acoustic cues to features

**DOI:** 10.1016/j.dib.2022.108275

**Published:** 2022-05-16

**Authors:** Maria-Gabriella Di Benedetto, Stefanie Shattuck-Hufnagel, Jeung-Yoon Choi, Luca De Nardis, Javier Arango, Ian Chan, Alec DeCaprio, Sara Budoni

**Affiliations:** a Research Laboratory of Electronics (RLE), Massachusetts Institute of Technology, Cambridge, MA 02139, USA; b Department of Information Engineering, Electronics and Telecommunications, Sapienza University of Rome, Via Eudossiana 18, Rome 00184, Italy; c Radcliffe Institute for Advanced Study, Harvard University, 10 Garden St, Cambridge, MA 02138, USA

**Keywords:** Speech processing, Lexical gemination, Syntactic gemination, Italian

## Abstract

The LaMIT database consists in recordings of 100 Italian sentences. The sentences in the database were designed so to include all phonemes of the Italian language, and also take into account the typical frequency of each phoneme in written Italian. Four native adult speakers of Standard Italian, raised and living in Rome, Italy, two female and two male, pronounced the sentences in two different recording sessions; two repetitions for each sentence per speaker were therefore collected, for a total of 800 recordings.

The database was specifically created for application in the LaMIT project, that focuses on the application to the Italian language of the Lexical Access model proposed by Ken Stevens for American English. The model relies on the detection of specific acoustic discontinuities called landmarks and other acoustic cues to features that characterize each phoneme. Each recording was thus processed to generate a set of labeling files that identify both predicted landmarks and other cues, and actual landmarks/cues. The labeling files, compiled according to the labeling syntax used in the Praat speech processing software, are also made available as part of the LAMIT database.

**Specifications Table**

**Table:** 

Subject	Signal Processing
Specific subject area	Acoustic analysis and landmark detection in the Italian language
Type of data	Waveform Audio File Format (WAV) files, TextGrid files
How data were acquired	Recorded using a Samson Meteor Mic USB microphone, in an Amplisilence recording booth by Amplifon. Acquired using the Audacity software tool with a sampling rate of 44.1 kHz and quantization set at 16 bits/sample.
Data format	Raw, Analyzed
Description of data collection	The set includes 100 sentences designed to reflect the typical frequency of appearance of phonemes in Italian. Sentences were recorded two times in two different recording sessions, leading to two repetitions for each sentence and for each speaker. The words to be pronounced were presented to the speakers on a laptop screen.
Data source location	Institution: Sapienza University of Rome City/Town/Region: Rome Country: Italy Data were collected in the Speech Communication Laboratory of the DIET Department, located in Via Eudossiana 18, 00184, Rome, Italy. Latitude: 41.893762939686034, Longitude: 12.493808281027881.
Data accessibility	Repository name: Mendeley Data Data identification number (permanent identifier, i.e. DOI number): 10.17632/sjwb9hymhn Direct link to the dataset: https://data.mendeley.com/datasets/sjwb9hymhn
Related research article	M.-G. Di Benedetto, S. Shattuck-Hufnagel, L. De Nardis, S. Budoni, J. Arango, I. Chan, and A. DeCaprio, ”Lexical and syntactic gemination in Italian consonantsDoes a geminate Italian consonant consist of a repeated or a strengthened consonant?” The Journal of the Acoustical Society of America, Volume 149, Issue 5, May 2021. DOI: 10.1121/10.0004987.

## Value of the Data

• The LAMIT database provides an exhaustive set of recordings of Italian sentences, uttered under controlled conditions, and allows for systematic acoustic analyses of the Italian language.
• The data are of interest to researchers in the fields of speech communication, speech processing, and speech recognition.
• The data can be used to investigate the lexical and syntactic gemination in Italian, and more in general all features related to vowels and consonants in running speech.
• A better understanding of distinctive phenomena in Italian, such as syntactic gemination, and other properties may help designing better performing automatic speech recognition systems for the Italian language.

## 1 Data Description

The database includes both raw data and analysed data. Raw data consist in audio recordings stored in .wav files, while analyzed data consist in .TextGrid text files that label each recorded sentence according to the multi-tiered labeling approach reflecting the Lexical Access model proposed by Stevens [1] for American English, and recently extended to the Italian language [2], foreseeing the following twelve labeling tiers:

• *Words* - boundaries between words in the sentence;
• *LEXI phoneme* - phonemes predicted on the basis on the phonetic representation of words and manually adjusted to take into account the actual waveform characteristics. Adjustments include both variations in the temporal placement of boundaries between phonemes and replacement of phonemes themselves, e.g. if a speaker pronounces an intervocalic /s/ as /z/, as common in several Italian dialects;
• *LM* - predicted landmarks based on phonemes;
• *LMMods* - modifications to landmarks taking into account the waveform characteristics, e.g. when a predicted landmark is missing;
• *vgplace* - predicted properties of vowels;
• *vgplace mods* - modifications to the properties of vowels based on waveform characteristics;
• *cplace* - predicted properties of consonants;
• *cplace mods* - modifications to the properties of vowels based on waveform characteristics;
• *nasal* - predicted properties of nasals;
• *nasal mods* - modifications to the properties of nasals based on waveform characteristics;
• *glottal* - predicted properties of glottal sounds;
• *glottal mods* - modifications to the properties of glottal sounds based on waveform characteristics.

Details on landmarks and other cues associated with each tier can be found in [2].

The data include 100 Italian sentences, designed so to take into account the natural frequency of phonemes in Italian, pronounced by 4 speakers; two repetitions were recorded for each speaker for each sentence, for a total of 800 sentences. These speech materials served in a recent analysis of lexical and syntactic gemination in Italian [3]. Each repetition of each sentence is associated with a wav file and a set of .TextGrid files containing the labels, that can be jointly opened with the Praat audio processing software [4], allowing both audio reproduction and visual inspection of waveform and labels. The database is organized in four folders, one for each speaker:

• SPEAKER_JV
• SPEAKER_LDN
• SPEAKER_MGDB
• SPEAKER_SB

Each folder contains two subfolders: one for audio recordings, named *Audio*, and one containing both .wav and .TextGrid files, named *Audio_and_labels*. Details on the content of each folder are provided below.

### 1.1 Audio folder

Each repetition of each sentence is stored in a separate wav file. Each file contains samples of a 1-channel recording, represented as 16 bits signed integers, with sampling rate 44.1 kHz. Files are named as follows:

<SPEAKER>_<SENTENCE>_v<REPETITION>.wav (e.g. JV_001_v1.wav, LDN_094_v2.wav, and so on).

### 1.2 Audio_and_labels folder

The *Audio_and_labels* folder contains a subfolder for each sentence, named from 001 to 100. Each subfolder contains, for each repetition, the .wav audio file and multiple .TextGrid files, providing information related to different subsets of the twelve tiers. In order to allow for an immediate understanding of the information contained in each .TextGrid file, a name tag was associated with each tier, and the tags associated with the tiers included in the file are appended to the file name, in the form:

<SPEAKER>_<SENTENCE>_v<REPETITION>_<TAG>_< TAG>⋯.TextGrid.

Table 1 provides a legend of the mapping between file name tags and tiers. Note that for landmarks, vowels, consonants, nasals and glottals, modifications are never provided in a .TextGrid file without the corresponding predictions; for example, the LMMods tier is never included without also including the LM tier. As a consequence, the file name tags for tiers including modifications are also used to indicate the presence of the tier providing the corresponding predicted properties. For instance, the file of speaker LDN, sentence 005, version 2 containing information on tiers LEXI phoneme, LM and LMMods is named LDN_005_v2_LEXI_LMm.TextGrid.

**Table 1:** Mapping between tier information and file name tags for .TextGrid files.

Tier	LEXI	LM	LMMods	vgplace	vgplace mods	cplace	cplace mods	nasal	nasal mods	glottal	glottal mods
**Tag**	LEXI	LM	LMm	VG	VGm	C	Cm	N	Nm	G	Gm

**Fig. 1:**
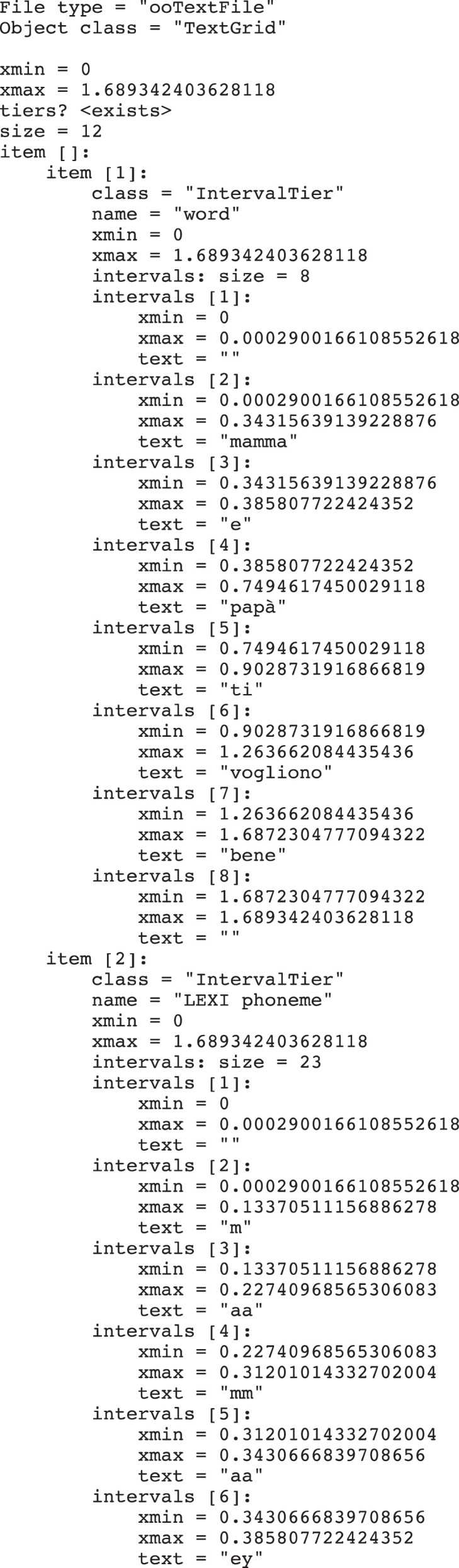
Excerpt from TextGrid file for sentence LDN_036_v1 showing the syntax adopted to define tiers. Tiers ’Word’ and ’LEXI phoneme’ are visible in the excerpt.

**Fig. 2:**
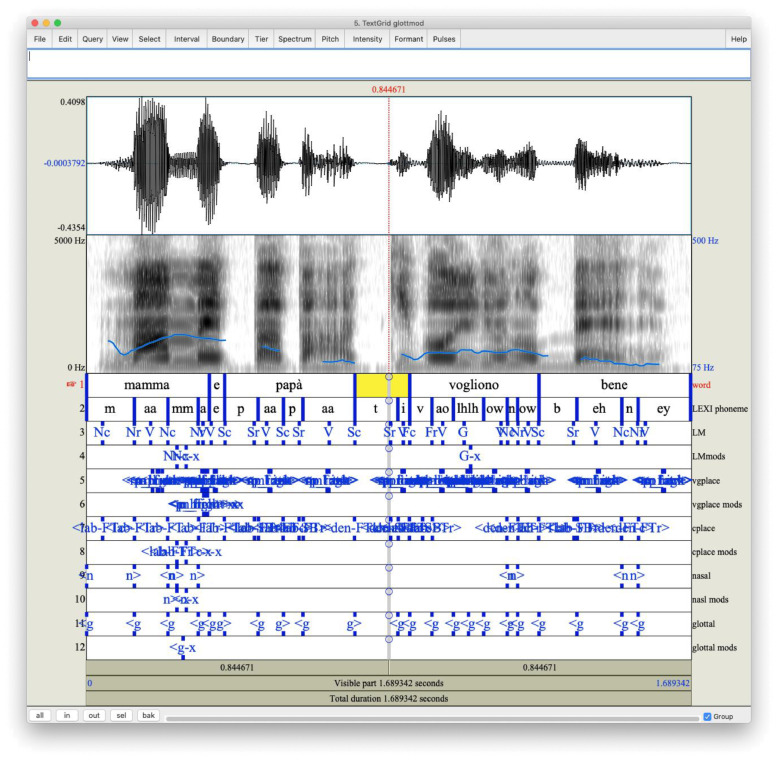
Example of Praat output for sentence LDN_036_v1, including waveform and corresponding spectrogram computed from the .wav file and labeling information stored in the TextGrid file.

Fig. 1 shows an example of the content of a .TextGrid file, structured according to the syntax rules defined in Praat, while Fig. 2 shows the combined representation of waveform and labeling information in Praat for one of the sentences in the dataset.

## 2 Experimental Design, Materials and Methods

Recording sessions were carried out in an Amplisilence recording booth by Amplifon, featuring internal sound absorbing panels to avoid voice reverberation, and characterized by an external noise reduction of about 30 dB at the frequencies of interest.

The microphone was an omnidirectional, monophonic Samson Meteor Mic USB microphone, with a flat frequency response between 20 Hz and 20 kHz and Signal to Noise Ratio of 96 dBA. The microphone was connected to an Apple MacBook Pro laptop running the Audacity software tool, set to single channel recording with a sampling rate of 44.1 kHz and quantization set at 16 bits/sample. Recordings were later exported as .wav files.

The laptop was placed inside the recording booth, allowing thus to keep the door of the booth closed during the recording sessions. Sentences were presented to the speaker by the operator on a second laptop placed in front of the glass window of the recording booth. The distance of the speaker’s lips from the microphone was monitored during the recording sessions and was kept at about 20 cm, by having the microphone hanging in front of the speaker at a height adjusted to match the height of speaker’s mouth. Four adult Italian native speakers raised and living in Rome (Italy), two men and two women aged from twenty-four to fifty, participated in the recordings. The speakers were pronunciation defectless and free of evident dialectal inflexions. Recording sessions were supervised by an acoustically trained person, in charge of pointing out evident mispronunciations and prompting a new recording when needed. Speakers were asked to maintain their natural speaking style in order to mitigate the impact of variations in emission levels and tempo.

## Ethics Statement

Informed consent was obtained from all subjects involved in the data measurement campaign. As no personal data is shared within the paper, ethics consent was not required.

The paper is not currently being considered for publication elsewhere.

## CRediT authorship contribution statement

**Maria-Gabriella Di Benedetto:** Writing – review & editing, Writing – original draft, Supervision, Conceptualization, Investigation, Methodology, Project administration. **Stefanie Shattuck-Hufnagel:** Writing – review & editing, Conceptualization, Investigation. **Jeung-Yoon Choi:** Writing – review & editing, Conceptualization, Investigation. **Luca De Nardis:** Data curation, Writing – original draft, Writing – review & editing, Software, Methodology, Visualization. **Javier Arango:** Data curation. **Ian Chan:** Data curation. **Alec DeCaprio:** Data curation. **Sara Budoni:** Data curation.

## Declaration of Competing Interest

The authors declare that they have no known competing financial interests or personal relationships that could have appeared to influence the work reported in this paper.

## Data Availability

The LaMIT database: a read speech corpus for acoustic studies of the Italian language toward lexical access based on the detection of landmarks and other acoustic cues to features (Mendeley Data).
